# Protection of Adipose Tissue by Pioglitazone in a Mouse Model of Doxorubicin Treatment

**DOI:** 10.1002/cbf.70120

**Published:** 2025-09-18

**Authors:** Loreana Sanches Silveira, Alexandre Abilio de Souza Teixeira, Luana Amorim Biondo, Luísa Menezes Silva, Luís Eduardo Duarte Gonçalves, Ana Beatriz Lima Pedroza, Niels Olsen Saraiva Camara, José Cesar Rosa‐Neto

**Affiliations:** ^1^ Immunometabolism Research Group, Department of Cell Biology and Development, Institute of Biomedical Sciences University of São Paulo (ICB1‐USP) São Paulo Brazil; ^2^ Department of Immunology Institute of Biomedical Sciences, University of São Paulo (ICB4‐USP) São Paulo Brazil

**Keywords:** doxorubicin, lipid metabolism, PPAR gamma, rosiglitazone, thiazolidinediones

## Abstract

Cancer is a global epidemic with increasing incidence, which needs continuous efforts to enhance treatment efficacy and reduce side effects. Our study focuses on the impact of doxorubicin, a widely used chemotherapeutic agent, on white adipose tissue (WAT) homeostasis and explores the potential mitigating effects of coadministration with pioglitazone, a peroxisome proliferator‐activated receptor‐gamma (PPARγ) activator. Using male C57BL/6 mice, we investigated the influence of doxorubicin and pioglitazone on WAT, considering factors such as weight loss, metabolic parameters, lipolysis, adipokines, and immune cell infiltration. Doxorubicin treatment resulted in weight loss, specifically affecting visceral adipose tissue, while coadministration with pioglitazone preserved inguinal adipose tissue (iWAT). Metabolic analyses revealed that doxorubicin induced hypoglycaemia, mitigated by pioglitazone, without significant effects on lipid profiles. Pioglitazone ameliorated the doxorubicin‐induced reduction in adiponectin, thereby contributing to the maintenance of glucose homeostasis. Lipolysis assays demonstrated doxorubicin‐induced lipotoxicity, particularly in (iWAT), which was attenuated by pioglitazone. Histological analysis showed no significant changes in adipocyte size, while flow cytometry revealed a reduction in pro‐inflammatory M1 macrophages in the co‐treated group. Based on gene expression profiles, pioglitazone appeared to modulate genes involved in lipid metabolism, with preliminary indications of a potential role in adipogenic processes. In summary, coadministration of pioglitazone during doxorubicin treatment appeared to attenuate alterations in WAT homeostasis associated with lipotoxicity. These findings contribute to the understanding of potential supportive strategies during doxorubicin‐based chemotherapy. Further studies should be conducted to define appropriate dosing regimens, treatment durations, and to evaluate potential effects on cancer progression and patient outcomes.

## Summary

Preserving adipose tissue homeostasis is critical for maintaining the quality of life in cancer patients. Although doxorubicin is an effective chemotherapeutic agent, it disrupts lipid metabolism and induces lipotoxicity. Our study demonstrates that coadministration of pioglitazone, a PPARγ agonist, mitigates these adverse effects by preserving inguinal adipose tissue, restoring glycaemic control, and reducing tissue inflammation without compromising the antitumor efficacy of doxorubicin. These findings are significant as they suggest a potential strategy to improve treatment tolerability, thereby positively impacting patient survival and quality of life. Furthermore, our work paves the way for the development of more effective and safer co‐therapies in clinical oncology, contributing to a more comprehensive approach to cancer care.

## 1 Introduction

Cancer is a major global health challenge, and its incidence has been steadily increasing [1]. Recent efforts to combat cancer have focused on mantaining patients' quality of life, improving pharmacological treatments and reducing chemotherapy side effects [2]. Cancer can be directly associated with the loss of strength, muscle, and white adipose tissue (WAT) mass and metabolic dysfunction, when cachexia occurs [3]. Furthermore, there are currently no approved treatments or drugs for cancer‐associated cachexia [4] and its prevalence in oncologic patients is approximately 30% [5].

Our group has demonstrated that chemotherapy can have a profound deleterious effect on adipose tissue (AT), leading to a loss of its ability to store triglycerides and produce adipokines [6]. The key regulator of gene transcription involved in glucose uptake, adipogenesis, lipogenesis, and adipokine production is the transcription factor peroxisome proliferator activated receptor gamma (PPARγ) [7].

Doxorubicin (DOXO), the main chemotherapeutic agent used in clinical practice against solid tumours either as a single agent or as part of a chemotherapy regimen, profoundly inhibits PPARγ [8]. This chemotherapeutic agent has been used in clinical practice since the 1960s due to its effectiveness against tumour cells [9]. However, its use is limited to low doses because of its toxicity [10]. This underscores the urgent need for co‐therapies that augment doxorubicin's therapeutic efficacy and/or attenuate its toxicity.

The PPAR family appears to be a key target in doxorubicin‐induced lipotoxicity. Therefore, it is highly relevant to evaluate whether animals subjected to a doxorubicin cycle with concomitant administration of pioglitazone (PIO), a PPARγ activator, can attenuate the chemotherapy‐induced lipotoxicity, thereby improving the quality of life and life expectancy of animals receiving the combination treatment.

Pioglitazone has primarily been studied in vitro and has shown positive effects on tumour cell growth [11, 12]. In vitro studies on PIO‐DOXO combination showed benefits in cell cycle regulation [13], migration [14], and apoptosis [13], while animal models highlighted the protective role of PIO against DOXO‐induced cardiac [15, 16] and cognitive [17, 18] side effects. The effects of PIO and DOXO, alone or combined, depend on tumour type [19], requiring further tissue‐specific studies. Importantly, among the tissues affected by DOXO, adipose tissue significantly influences overall health and quality of life, making it a crucial target for supportive interventions.

Building on these findings, the primary objective of our study is to determine whether the co‐therapy of chemotherapy and pioglitazone can mitigate the deleterious effects of doxorubicin on AT homeostasis, as well as its potential impact on tumour progression and animal survival.

## 2 Methods

### 2.1 Animals

Approximately 8 weeks old male C57BL/6 mice were housed in an animal facility room with a 12–12 h light‐dark cycle and a temperature of 23 ± 2°C. Each cage contained 3 to 4 animals. They were fed a standard diet (Nuvital from Nuvilab®, Colombo, PR) and water ad libitum. All procedures in this study followed ethical principles of animal experimentation under the Animal Experimentation Ethics Committee of the University of São Paulo CEUA N° 9686120320.

### 2.2 Tumour Cell Inoculation

Lewis lung carcinoma (LLC) cells were grown in Dulbecco's Modified Eagle's Medium (DMEM; GIBCO, Invitrogen, NY) containing 10% fetal bovine serum (FBS; Atlanta Biologicals, Lawrenceville, GA), 100 U/mL penicillin, and 100 µg/mL streptomycin. Cultures were maintained at 37°C in a humidified incubator with 5% CO₂. For tumour implantation, 5 × 10⁵ viable cells, resuspended in sterile 0.9% saline, were administered inguinally into the right flank of each mouse. Cell viability was confirmed by Trypan Blue exclusion assay.

### 2.3 Experimental Protocol

Mice were divided into four groups (*n* = 4–5 each): a) control group, b) control + pioglitazone group (PIO), c) doxorubicin group (DOXO), and d) doxorubicin + pioglitazone group (DOXO + PIO). Our experimental design follows a standard pharmacological approach to evaluate the individual and combined effects of the tested drugs. This design allows us to determine the individual effects of each drug while avoiding confounding factors, examine possible interactions when both drugs are administered together, and ensure scientific rigor and comparability, as this approach is widely used in pharmacological studies with animal models. The DOXO group received a weekly dose for 5 weeks (3 mg/kg of body weight intraperitoneally), while the control animals received the same volume of saline [20]. In addition, the mice received daily gavage with pioglitazone (10 mg/kg of body weight) or saline for the duration of the 5‐week doxorubicin treatment. Animals received gavage and/or intraperitoneal DOXO at the same time of the day during the first hours of the light cycle. At the end of the experimental protocol, the animals were euthanized by isoflurane inhalation followed by decapitation after 6 h fasting. WAT depots (inguinal, epididymal, and retroperitoneal) were removed, weighed, frozen in liquid nitrogen, and stored at −80°C. Blood were centrifuged at 600 g for 15 min, and the serum was collected and frozen at −80°C for further analysis.

### 2.4 Histological Analysis and Measurement of Adipocyte Diameter

AT depots were fixed in paraformaldehyde 4% and embedded in paraffin. Two sections of 5 µm were taken per block, stained with haematoxylin and eosin, and imaged at 40X magnification. The images were further used to adipocytes quantification and measurements using Adiposoft software available at http://sw.wikkii.com/wiki/Adiposoft according to Galarraga (2012) [21].

### 2.5 Characterization of the Subpopulation of Macrophage Infiltrates in Inguinal Adipose Tissue by Flow Cytometry

For the isolation of infiltrated cells, iWAT was incubated with collagenase type 2 (1.5 mg/mL; Sigma‐Aldrich®) diluted in RPMI medium for 1.5 h at 37°C. The suspension was filtered using a 100‐micrometer cell strainer and centrifuged at 200 g for 10 min at 4°C. The pellet was resuspended in red blood cell lysis buffer and centrifuged again for 10 min at 500 g at 4°C. The supernatant was discarded, and the cells were resuspended in PBS supplemented with 2% foetal bovine serum. For macrophage identification, fluorescent monoclonal antibodies for the following markers were used: CD45 (PercP Cy‐7), F4/80 (APC Cy‐7), CD11b (Pacific Blue), CD206 (PE), Cd11c (PE‐Cy‐7), Ly6G (APC), and Ly6G (FITC) from BD Biosciences®. Within the macrophage population, the CD80 + CD206‐ subpopulations were considered M1, and the CD206 + CD80‐ subpopulations were considered M2. For identification of resident macrophages, the Ly6C+ subpopulations were considered, while Ly6C‐ represented nonresident macrophages. Gatting strategy is shown in Supporting Information Figure 3.4. Sample acquisition was performed on the FACS ARIA II flow cytometer (BD, Beckton Dickson, NJ, USA), and data analysis was conducted using FlowJo 9.5.3 software (Treestar®).

### 2.6 Lipolysis Assay

Lipolysis was estimated by measuring the release of glycerol in the incubation medium. Fresh AT slices from retroperitoneal and inguinal depots were incubated in buffer for 30 min at 37°C in the presence or absence of isoproterenol (10 µM) [22]. The incubation medium was collected to assess the released glycerol (Free glycerol determination kit; Sigma®).

### 2.7 Biochemical Assays

The concentrations of total cholesterol and triacylglycerol were determined using enzymatic kits according to the manufacturers' instructions (Labtest®, Lagoa Santa, MG, Brazil). Nonesterified fatty acids in the serum were assessed by colorimetric assay (NEFA‐HR, Wako®, Mountain View, CA, USA).

### 2.8 Gene Expression by Real‐Time PCR

The quantification of gene expression was performed using the comparative ΔΔCt method, with B2m expression as the internal standard. Total RNA was extracted using Trizol® (Invitrogen Life Technologies, Carlsbad, USA), and reverse‐transcribed to cDNA using a High‐Capacity cDNA kit (Applied Biosystems, Warrington, UK). Genes involved in WAT metabolism and inflammation were determined (FAS, PGC‐1alpha, SREBP‐1, PPARγ 1 and 2, CPT‐1, LPL, and NFkB) Table 1.

**Table 1:** Primer sequences for real‐time PCR.

Gene	5′‐Forward Primer‐3′	5′‐Reverse Primer‐3′
*B2m*	TTCTGGTGCTTGTCTCACTGA	CAGTATGTTCGGCTTCCCATTC
*FAS*	GATTCGGTGTATCCTGCTGTC	CATGCTTTAGCACCTGCTGT
*LPL*	GTCTCGCTGACACTGGACAAA	CCCACTTTCAAACACCCAAA
*Pparg1*	ATCTTAACTGCCGGATCCAC	CAAACCTGATGGCATTGTGAG
*Pparg2*	ATCTTAACTGCCGGATCCAC	CAAACCTGATGGCATTGTG
*Srebp1c*	TGGACCACAGAAAGGTGGA	ATGGCCTTGTCAATGGAACT
*PGC1α*	GCTCAAGCCAAACCAACAA	CCACACTTAAGGTTCGCTCA
*NFκb*	AGGCTTCTGGGCCTTATGTG	TGCTTCTCTCGCCAGGAATAC

### 2.9 Enzyme‐Linked Immunosorbent Assay (ELISA)

Inguinal adipose tissue samples (80–100 mg) were homogenized in RIPA buffer (0.625% Nonidet P‐40, 0.625% sodium deoxycholate,6.25 mM sodium phosphate, and 1 mM ethylenediaminetetraacetic acid at pH 7.4) containing 10 ug/mL of protease inhibitor cocktail Roche®. The samples were centrifuged at 20,000 g, 4°C, for 30 min, and the supernatants used for protein quantification using the Bradford assay (Bio‐Rad, Hercules, California). The supernatants were used to determine the protein levels of adiponectin, IL‐1β, IL‐4, IL‐6, IL‐10, TNF‐α, VEGF, and MCP‐1 by ELISA (DuoSet ELISA, R&D Systems, Minneapolis, MN).

### 2.10 Statistical Analysis

Data were analysed using GraphPad Prism statistics software package version 9.0 for Windows (GraphPad Software, San Diego, CA, USA). The data were presented as mean ± standard error (SE). The normality of the data was confirmed by the Kolmogorov–Smirnov test and differences among the groups was performed by One‐way ANOVA followed by *Fisher's LSD* posttest. A linear regression model was used to assess the relationship between drugs combination by using the coefficient of determination (R²). A value of *p* < 0.05 was considered statistically significant.

## 3 Results

### 3.1 Pioglitazone Did Not Prevent Visceral Adipose Tissue Loss in Doxorubicin Treated Mice

**Figure 1:**
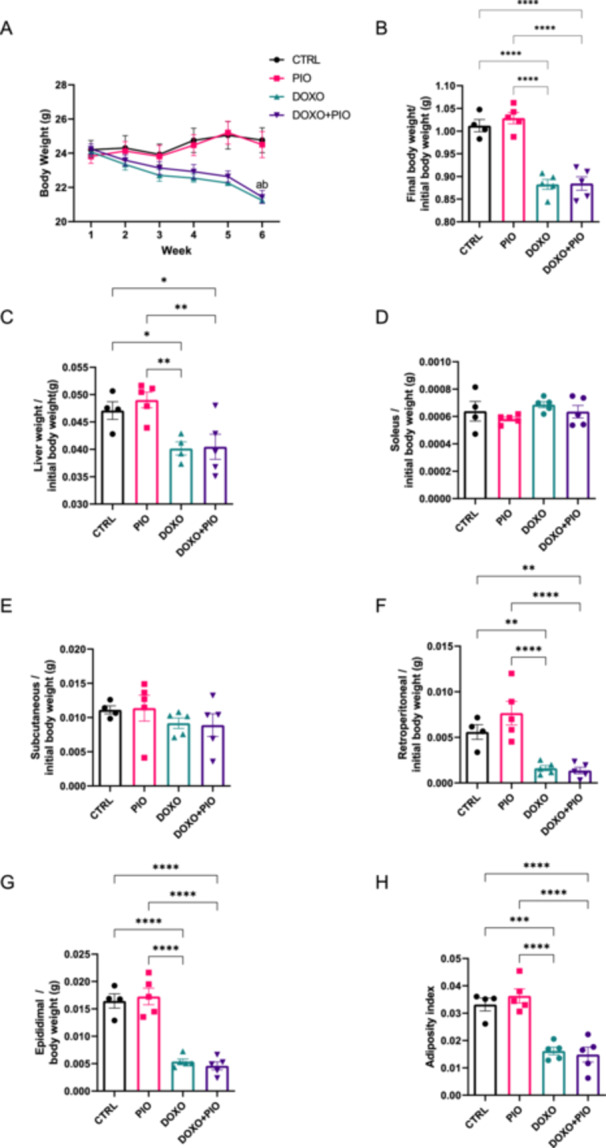
Total body weight (BW) and body composition of mice treated with doxorubicin (3 mg/kg weekly) and/or pioglitazone (10 mg/Kg daily gavage) or saline for 6 weeks. Mean and standard error (SE) of Total body weight over the weeks (A); Body weight at the end of the protocol (B); liver and BW ratio (C), soleus and BW ratio (D), inguinal adipose tissue (E) retroperitoneal adipose tissue (F) epididimal adipose tissue (G) and the sum of all white adipose fat pads [adiposity index] (H). ANOVA followed by Fisher's LSD posttest. *p ≤ 0.05; **p ≤ 0.01; ***p ≤ 0,001; a = different from CTRL, b = different from PIO, DOXO= doxorubicin, PIO = pioglitazone.

Doxorubicin weight loss was not prevented by PIO [*r* ^2^ = 0.883 and *p* < 0.001] (Figure 1A,B) and it seems to be related to AT [*r* ^2^ = 0.823 and *p* < 0.001] and liver [*r* ^2^ = 0.593 and *p* = 0.004] weight reduction (Figure 1C,F–H). Inguinal AT was preserved in the DOXO and DOXO + PIO groups, although RET [*r* ^2^ = 0.751 and *p* < 0.001] and EPI [*r* ^2^ = 0.892 and *p* < 0.001] were affected by the chemotherapy and PIO was not able to avoid the loss. Additionally, at this dose, 5 weeks treatment DOXO did not induced muscle mass loss [*r* ^2^ = 0.189 and *p* = 0.35] (Figure 1D).

### 3.2 Pioglitazone was Efficient to Avoid Hypoglycaemia and Restored Adiponectin Levels in Doxo‐Treated Mice

**Figure 2:**
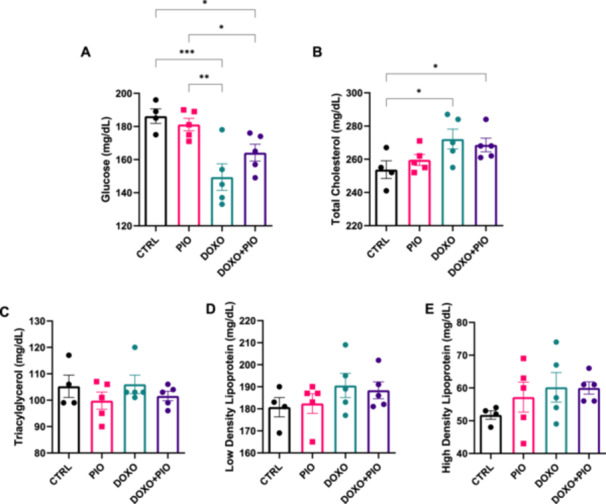
Metabolic variables from serum of mice treated with doxorubicin (3 mg/kg weekly) and/or pioglitazone (10 mg/kg daily gavage) or saline for 6 weeks. Mean and standard error (SE) of glucose (A), total cholesterol (B), triacylglycerol (C), low density lipoprotein LDL (D) and high density lipoprotein HDL(E). ANOVA followed by Fisher's LSD posttest. *p ≤ 0.05; **p ≤ 0.01; ***p ≤ 0,001. DOXO = doxorubicin, PIO = pioglitazone.

Among the metabolic variables analysed in serum, glycaemia and cholesterol were the only ones affected by DOXO treatment. Animals treated with the chemotherapy had lower fasting glucose levels, which was restored by the concomitant PIO treatment [*r* ^2^ = 0.631 *p* = 0.0015] (Figure 2A). Total cholesterol was elevated in DOXO treated and the insulin sensitizer PIO did not influence in its level (Figure 2B). For all the other lipid related metabolic variables measured, such as triacylglycerol, HDL, and LDL, neither treatment had considerable effect (Figure 2C–E)

**Figure 3:**
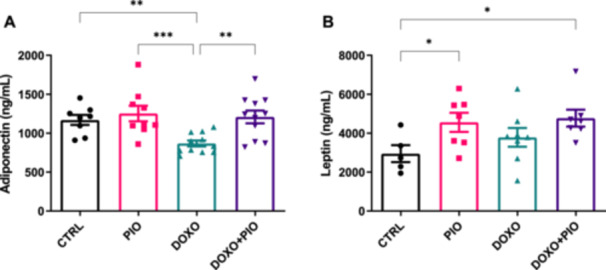
Adipokines from serum of mice treated with doxorubicin (3 mg/kg weekly) and/or pioglitazone (10 mg/kg daily gavage) or saline for 6 weeks. Mean and standard error (SE) of (A) adiponectin and (B) leptin, assessed by ELISA kits. ANOVA followed by Fisher's LSD posttest. *p ≤ 0.05; **p ≤ 0.01; ***p ≤ 0,001. DOXO = doxorubicin, PIO = pioglitazone.

Our results also demonstrated that DOXO treatment reduced the concentration of adiponectin in the serum, and co‐treatment with PIO was able to mitigate this doxorubicin‐induced effect [*r* ^2^ = 0.342 and *p* = 0.0019] (Figure 3A). On the other hand, Leptin did not appear to be affected by the chemotherapeutic drug, but it was slightly exacerbated by PIO, alone or with DOXO (Figure 3B).

### 3.3 Pioglitazone Attenuated Basal Lipolysis in Sub at From Dox‐Treated Mice With no Changes in Its Morphology

**Figure 4:**
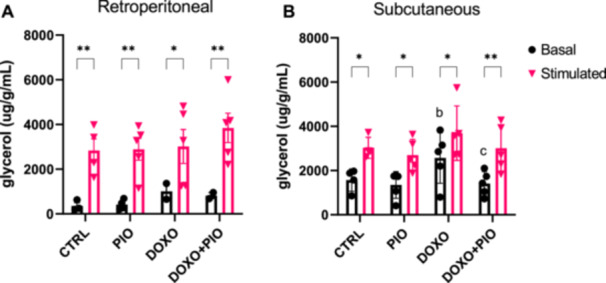
Glycerol concentration from adipose tissue of mice treated with doxorubicin (3 mg/kg weekly) and/or pioglitazone (10 mg/Kg daily gavage) or saline for 6 weeks. Retroperitoneal adipose tissue (A) and Inguinal adipose tissue (B) were stimulated or not with isoproterenol (10 mM) and glycerol content in the buffer was measured. Two‐way ANOVA followed by Fisher's LSD post test *p ≤ 0.05; **p ≤ 0.01; b = different from PIO basal, c = different from DOXO basal, DOXO = doxorubicin, PIO = pioglitazone.

Lipolytic activity was assessed in both inguinal and retroperitoneal adipose depots using isoproterenol (10 mM). As expected by its more lipolytic basal physiology, iWAT showed to be more sensitive to the lipolysis induced by DOXO treatment, which was restored by PIO (Figure 4B). The visceral AT (retroperitoneal) lipolysis did not seem to be influenced by PIO or DOXO (Figure 4A).

**Figure 5:**
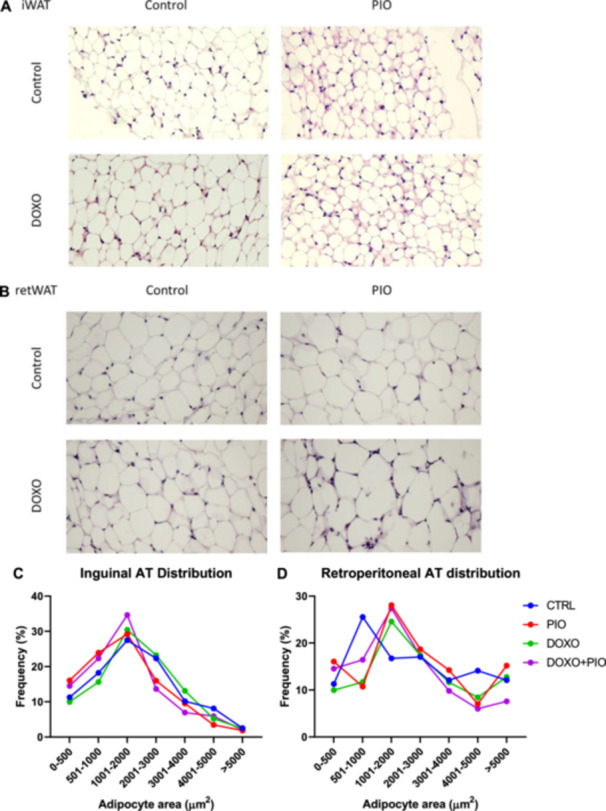
White adipose tissue histology and adipocyte area distribution tissue of mice treated with doxorubicin (3 mg/kg weekly) and/or pioglitazone (10 mg/kg daily gavage) or saline for 6 weeks. Inguinal (A) and Retroperitoneal (B) fat depots were morphologically analysed using eosin and haematoxylin staining (40X magnification) and adipocytes area frequencies (C and D) were distributed by size (µm^2^). Adiposoft® were used for adipocyte area quantification. ANOVA followed by Fisher's LSD post test *p* < 0.005. DOXO = doxorubicin, PIO = pioglitazone.

Inguinal AT had no differences in adipocyte size and/or distribution among the treatments (Figure 5D) while retroperitoneal AT had a more heterogeneous distribution (Figure 5C).

### 3.4 Pioglitazone Attenuated Adipose Tissue Pro‐Inflammatory Macrophages Infiltration in Doxorubicin Treated Mice

Our next step was to investigate if there were any differences on inflammatory status among the treatments. For this, we started analysing immune cells infiltration in iWAT. In this figure, the presented data from flow cytometry, allowing us to assess macrophage infiltration, as well as whether they exhibited markers on their surface indicative of classical (Cd11c^+^) or alternative (CD206^+^) macrophage phenotypes.

**Figure 6:**
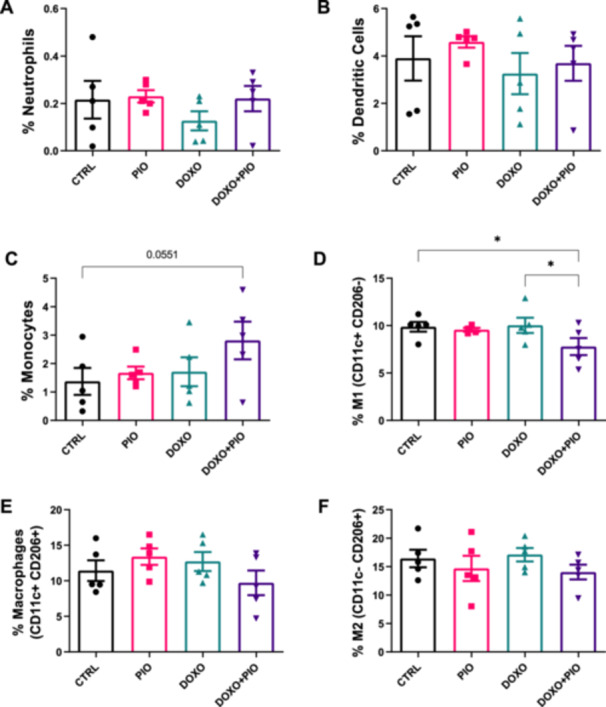
Inguinal adipose tissue immunophenotyping by flow cytometry tissue of mice treated with doxorubicin (3 mg/kg weekly) and/or pioglitazone (10 mg/kg daily gavage) or saline for 6 weeks. Neutrophils (A) dendritic cells (B) Monocytes (C) M1 macrophages (D), M2 macrophages (E) and pro‐inflammatory (F) cells frequency were calculated from total leucocytes (CD45 + ). ANOVA followed by Fisher's LSD post test *p ≤ 0.05 (*n* = 5). DOXO = doxorubicin, PIO = pioglitazone.

There was reduction in the frequency of M1 macrophages in animals receiving DOXO + PIO compared to those that received saline (CTRL) or only DOXO (Figure 6D) despite a tendency in augmented monocytes frequency in the combined treatment when compared to saline [CTRL vs. DOXO + PIO *p* = 0.055] (Figure 6C). Still, we did not observe any relevant difference in the phenotype of others immune cells infiltrated in AT (Figure 6A,B,E and F). Cytokine content on iWAT was not modulated by DOXO and/or PIO treatments (Supporting Information Figure 3.4).

**Figure 7:**
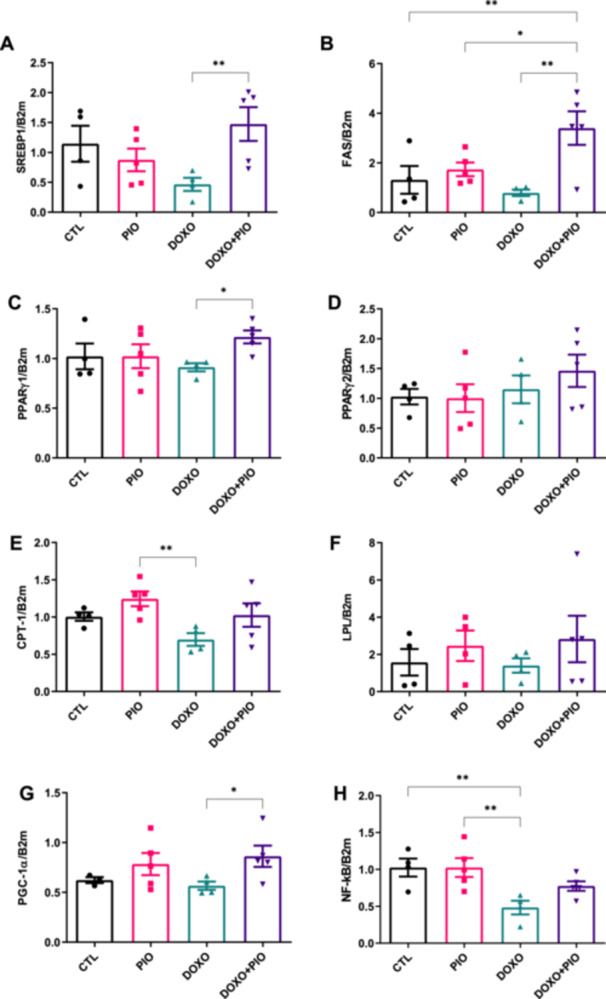
Expression of genes associated with lipid metabolism and inflammation in inguinal adipose tissue. Sterol regulatory element‐binding protein 1 [SREBP‐1] (A), fatty acid synthase [FAS] (B), Peroxisome proliferator‐activated receptor γ1 [PPARg1] (C), PPARG2 (D) Carnitine palmitoyl transferase I [CPT‐1] (E) Lipoprotein lipase [LPL] (F) Peroxisome proliferator‐activated receptor‐gamma coactivator (PGC)−1alpha [PGC1a] (G) Nuclear factor kappa‐light‐chain‐enhancer of activated B cells [NFκB] (H) gene expression by ΔΔCt was assessed via quantitative PCR to investigate the transcriptional expression of the genes. Data is expressed as the gene and housekeeping gene ratio. ANOVA one way followed by Fisher posttest *p ≤ 0.05 (*n* = 5). DOXO = doxorubicin, PIO = pioglitazone.

Protein abundance of cytokines (Supporting Information Figure 3.4) and gene expression of genes related to lipid metabolism were also evaluated. Despite the reduced gene expression of pro‐inflammatory transcriptional factor NFkB in the doxorubicin‐treated animals [*r* ^2^ = 0.549 and *p* = 0.009] (Figure 7H), no significant differences were found in the content of its gene related: IL‐1β (Supporting Information Figure 3.4). Furthermore, as expected, we observed a PIO‐mediated increase in SREBP‐1 [*r* ^2^ = 0.407 and *p* = 0.056], FAS [*r* ^2^ = 0.548 and *p* = 0.009], and PGC1α [*r* ^2^ = 0.327 and *p* = 0.148] genes expression in DOXO + PIO treated animals (Figure 7 A,B and G) compared to DOXO only.

### 3.5 Co‐Treatment With Pioglitazone Did Not Affect Survival in Tumour‐Bearing Mice

**Figure 8:**
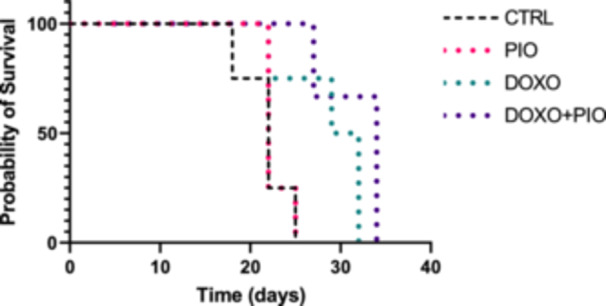
Kaplan Meier survival curve of tumour‐bearing mice treated with doxorubicin (3 mg/kg weekly) and/or pioglitazone (10 mg/Kg daily gavage) or saline for 6 weeks. DOXO = doxorubicin, PIO = pioglitazone.

Lastly, to understand the concomitant effect of the drugs on life expectancy, animals were injected with LLC tumour and submitted to the same treatments. The groups treated with doxorubicin alone or in combination with pioglitazone had increased life span (Figure 8), suggesting that PIO treatment did not impact the chemotherapeutical effect of DOXO, although body weight was preserved in DOXO + PIO group (Supporting Information Figure 3.4).

## 4 Discussion

**Figure 9:**
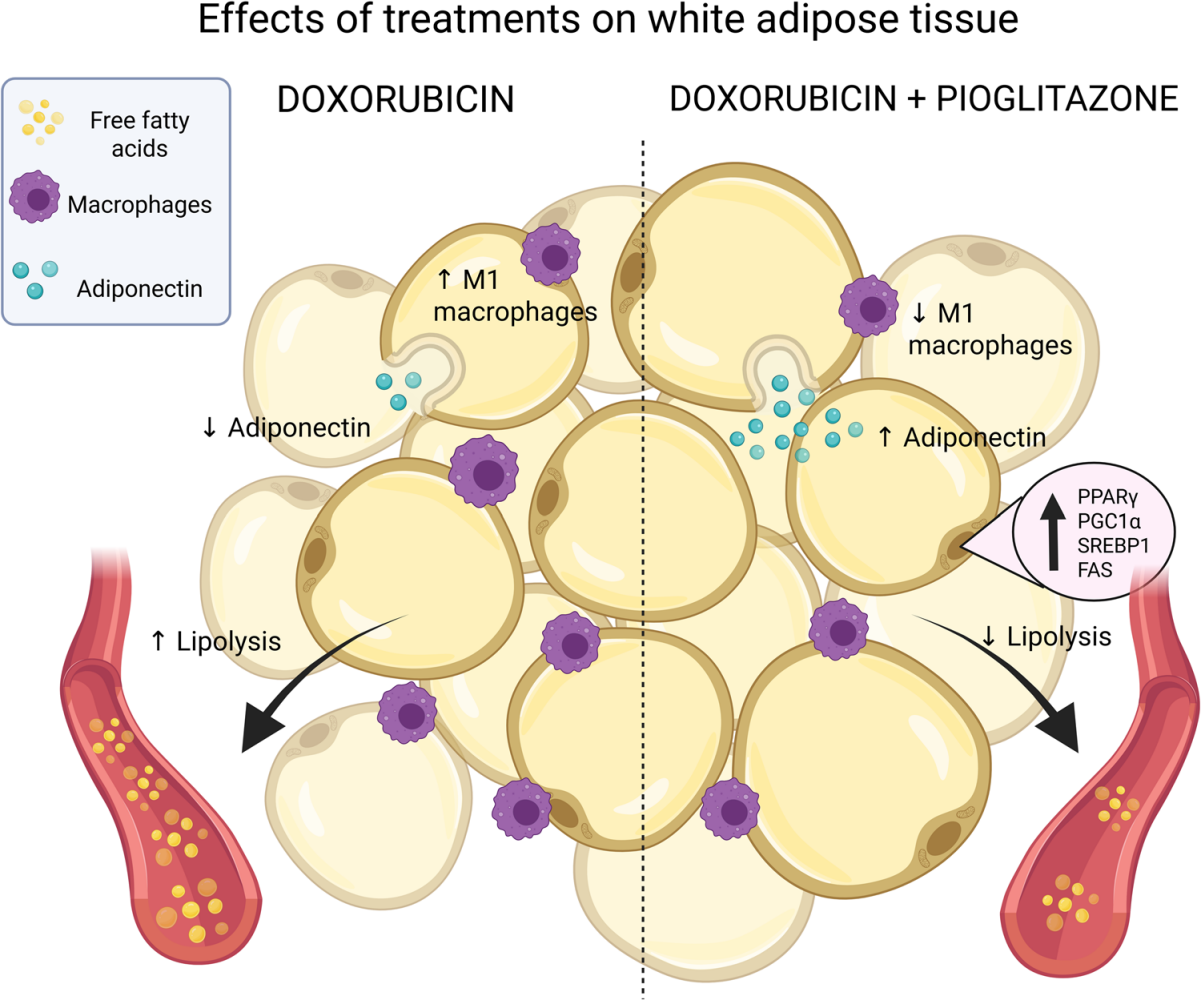
Administration of pioglitazone during doxorubicin treatment can alleviate the lipotoxic effects of this chemotherapy drug without affecting life span. This reduction in the impact of the insulin sensitizer treatment on adipose tissue homeostasis may enhance the quality of life for patients undergoing doxorubicin therapy. *Created with* BioRender.com.

Doxorubicin treatment reduced weight gain, visceral adipose mass, and caused significant metabolic disturbances. In contrast, co‐treatment with PIO provided greater protection to iWAT (Figure 9), consistent with our previous findings [6, 23]. Furthermore, increased basal lipolysis suggests a lipotoxic effect of doxorubicin, which was mitigated by PIO in iWAT.

Notably, doxorubicin accumulates in AT of both mice and humans 72 h after intravenous infusion, directly effecting the tissue [24]. In oncology patients, adipose tissue loss is a marker of poor prognosis and increased risk. Preservation of adipose mass could therefore contribute to improved survival and quality of life [25]. The association between the fat depots loss and worsening clinical outcomes is linked to endocrine function to AT. Furthermore, PPARγ activity play a key role in adipogenesis, particularly in iWAT. Reduced PPARγ activation impairs the expansion capacity of AT, leading to ectopic lipid storage in organs such as the liver, skeletal muscle, and heart, thereby promoting lipotoxicity [26]. In our study, PIO coadministration preserved inguinal adipose mass, highlighting its potential clinical relevance.

Regarding the expression of genes related to inflammation and lipid metabolism, ours results align to previously published data, showing reduced NFkB and CPT‐1 expression by DOXO treatment [6, 23]. DOXO‐treated mice exhibited lower SREBP1, FAS, and PGC1α expression than the DOXO + PIO group, suggesting PPARγ‐mediated restoration of adipogenesis. It is possible that prolonged treatment or higher DOXO doses would lead to further weight reduction, as observed in the retroperitoneal AT. Previous studies indicate that DOXO inhibits adipogenesis and impairs adipocyte glucose and lipid uptake [27].

We also observed a reduction in the frequency of infiltrated M1 macrophages only in animals treated with both drugs. This finding is particularly relevant because other groups have demonstrated a reduction in AT inflammation in animals treated with PIO [28, 29]. It has been proposed that PIO may stimulate macrophage differentiation into the M2 phenotype through a PPARγ‐dependent pathway, leading to reduced inflammation [30, 31]. Even a reduction in macrophages infiltration alone can lower AT inflammation and type 2 diabetes risk [32]. In our study, pro‐inflammatory CD11c + CD206‐ AT macrophages tended to be lower in DOXO + PIO treated mice. This specific subset of macrophages is associated with insulin resistance in human obesity [33]. The literature supports PPAR γ activation as potential anti‐inflammatory pathway, with pioglitazone increasing the M2/M1 macrophage ratio [34], reducing pro‐inflammatory cytokines production [35], and elevating adiponectin and IL‐10 levels while decreasing TNF‐α in AT [36].

In our study, pioglitazone increased adiponectin, IL‐6, and IL‐10 in iWAT but did not alter TNF‐α or IL‐1β levels. Interestingly, unlike in conditions characterized by low‐grade inflammation such as obesity and type 2 diabetes [37, 38, 39], PPARγ activation in our model did not lead to a reduction in classical pro‐inflammatory cytokines This may be explained by the absence of low‐grade inflammation following DOXO treatment in our experimental setting.

Adiponectin, an adipokine exclusively expressed by adipocytes and strongly regulated by PPARγ, was reduced in the circulation of DOXO‐treated animals, whereas PIO co‐treatment restored its serum levels. Adiponectin enhances insulin sensitivity in peripheral tissues and the liver, and modulates insulin synthesis and secretion by pancreatic β‐cells [40, 41, 42, 43]. Thus, PIO coadministration may counteract DOXO's detrimental effects on glucose homeostasis [44]. Notably, recent have reported additional benefits of this drug combination beyond AT metabolism, including cardioprotection [15] and neuroinflammation attenuation [18].

A limitation of our study is the marked reduction of retroperitoneal AT in DOXO‐treated animals, which prevented additional analyses (e.g., flow cytometry and gene and protein expression) in both depots. Thus, further analyses were performed only in subcutaneous inguinal AT. Although transcriptional changes do not always reflect protein‐level or physiological effects, our data provide initial insights into molecular processes modulated by the treatments. The absence of protein measurements is acknowledged as a limitation to be addressed in future studies.

This study was designed as an exploratory investigation to evaluate the effects of co‐therapy on AT homeostasis in mice. We did not perform an a priori sample size calculation. We acknowledge that the relatively small group sizes (*n* = 4–5) may limit the statistical power and generalizability of the results. However, this sample size is comparable to previous studies using murine models to analyse AT responses under similar experimental conditions [45]. Future studies with larger cohorts are warranted to confirm and extend these findings.

Previous studies have shown that DOXO can inhibit PPARγ in a dose‐dependent manner [27]; the dose used in the present study was sufficient to elicit this effect. We acknowledge that the small sample size may have limited our ability to detect improvements in survival among tumour‐bearing animals treated with DOXO in combination with PIO. Nonetheless, the absence of any survival detriment with coadministration supports further investigation in larger preclinical studies. Preservation of WAT mass induced by PIO treatment can potentially enhance the quality of life and survival, warranting additional studies on tumour progression.

Under our experimental conditions, the concomitant administration of pioglitazone did not alter tumour volume or weight compared with doxorubicin alone. While this finding should be interpreted with caution given the exploratory nature of the study, it can be viewed as a favourable outcome, indicating that the potential protective effects of pioglitazone on adipose tissue homeostasis were not accompanied by a reduction in the antitumor efficacy of doxorubicin.

In conclusion, our findings suggest that pioglitazone coadministration during doxorubicin treatment may attenuate chemotherapy‐induced lipotoxic effects on adipose tissue, without affecting life span under the present experimental conditions. While these results provide preliminary evidence of a protective role for pioglitazone in adipose tissue homeostasis, further studies with larger sample sizes, diverse tumour models, and extended follow‐up are required to confirm and expand these observations, ultimately assessing their translational relevance to clinical practice.

## Conflicts of Interest

The authors declare no conflicts of interest.

## Supporting information


**Figure S1:** Total body weight (BW) and body composition of tumour‐bearing mice treated with doxorubicin (3 mg/kg weekly) and/or pioglitazone (10mg/Kg daily gavage) or saline for 6 weeks. Mean and standard error (SE) of (A) Body weight at the end of the protocol; (B) tumour weight at the end of the protocol; (C) tumour volume at the end of the protocol; (D) retroperitoneal adipose tissue; (E) epididymal adipose tissue (F) subcutaneous adipose tissue. ANOVA followed by Fisher's LSD post‐test. *p ≤ 0.05; **p≤0.01; ***p ≤ 0,001; DOXO = doxorubicin; PIO = pioglitazone. **Figure S2:** Subcutaneous adipose tissue cytokine content from mice treated with doxorubicin (3 mg/kg weekly) and/or pioglitazone (10mg/Kg daily gavage) or saline (CTRL) for 6 weeks. **Figure S3:** Gating strategy of subcutaneous adipose tissue immunephenotyping by flow cytometry tissue of mice treated with doxorubicin (3 mg/kg weekly) and/or pioglitazone (10mg/Kg daily gavage) or saline for 6 weeks.

## Data Availability

The datasets generated and analysed during the current study are available from the corresponding author upon request.
