# Supplementary material for: Protection of Adipose Tissue by Pioglitazone in a Mouse Model of Doxorubicin Treatment
Source: Cell Biochem Funct. 2025 Sep 18;43(9):e70120. doi: 10.1002/cbf.70120 (PMC12446893; doi:10.1002/cbf.70120)
Supplement: Supplementary file 1 — Figure S1: Total body weight (BW) and body composition of tumour‐bearing mice treated with doxorubicin (3 mg/kg weekly) and/or pioglitazone (10mg/Kg daily gavage) or saline for 6 weeks. Mean and standard error (SE) of (A) Body weight at the end of the protocol; (B) tumour weight at the end of the protocol; (C) tumour volume at the end of the protocol; (D) retroperitoneal adipose tissue; (E) epididymal adipose tissue (F) subcutaneous adipose tissue. ANOVA followed by Fisher's LSD post‐test. *p ≤ 0.05; **p≤0.01; ***p ≤ 0,001; DOXO = doxorubicin; PIO = pioglitazone. Figure S2: Subcutaneous adipose tissue cytokine content from mice treated with doxorubicin (3 mg/kg weekly) and/or pioglitazone (10mg/Kg daily gavage) or saline (CTRL) for 6 weeks. Figure S3: Gating strategy of subcutaneous adipose tissue immunephenotyping by flow cytometry tissue of mice treated with doxorubicin (3 mg/kg weekly) and/or pioglitazone (10mg/Kg daily gavage) or saline for 6 weeks. [file CBF-43-e70120-s001.docx]

SUPPLEMENTAL MATERIAL

Figure S 1. Total body weight (BW) and body composition of tumour-bearing mice treated with doxorubicin (3 mg/kg weekly) and/or pioglitazone (10mg/Kg daily gavage) or saline for 6 weeks. Mean and standard error (SE) of (A) Body weight at the end of the protocol; (B) tumour weight at the end of the protocol; (C) tumour volume at the end of the protocol; (D) retroperitoneal adipose tissue; (E) epididymal adipose tissue (F) subcutaneous adipose tissue. ANOVA followed by Fisher`s LSD post-test. *p ≤ 0.05; ** = p≤0.01; *** p ≤ 0,001; DOXO= doxorubicin; PIO = pioglitazone.

Figure S 2. Subcutaneous adipose tissue cytokine content from mice treated with doxorubicin (3 mg/kg weekly) and/or pioglitazone (10mg/Kg daily gavage) or saline (CTRL) for 6 weeks. Mean and standard error (SE) of (A) TNF-alfa, (B) IL-1beta, (C) IL-6, (D) MCP-1, (E) IL-4, (F) IL-10, (G) VEGF and (H) Adiponectin, assessed by ELISA kits. and normalized by adipose tissue protein. ANOVA followed by Fisher`s LSD post-test. *p ≤ 0.05; ** = p≤0.01; *** p ≤ 0,001. DOXO= doxorubicin; PIO = pioglitazone.


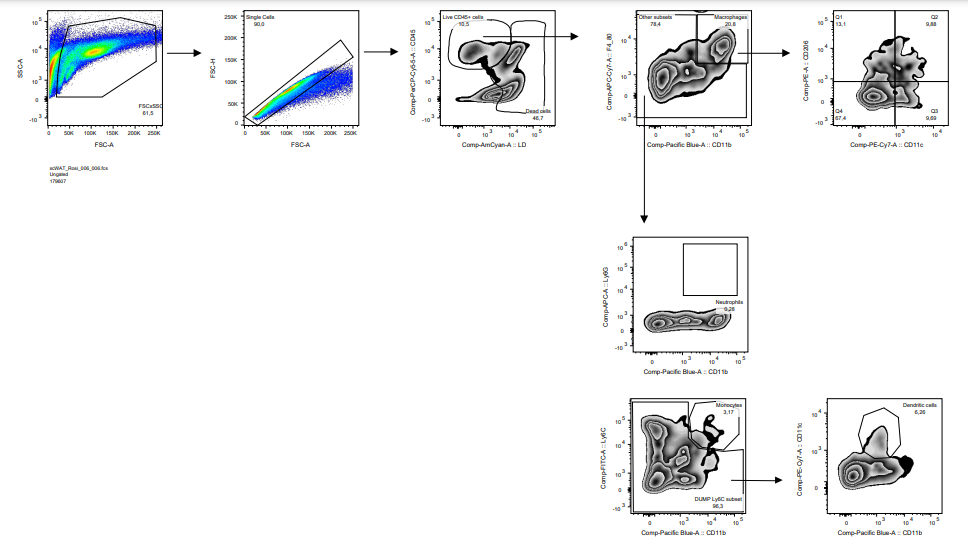


Figure S 3. Gating strategy of subcutaneous adipose tissue immunephenotyping by flow cytometry tissue of mice treated with doxorubicin (3 mg/kg weekly) and/or pioglitazone (10mg/Kg daily gavage) or saline for 6 weeks.
